# Naturally occurring radionuclides (NOR) and rare earth elements (REE) in soil and cereals in a high background area, Fen complex, Norway

**DOI:** 10.1007/s00411-026-01222-x

**Published:** 2026-05-04

**Authors:** H Haanes, R Gjelsvik, J Hoftuft, J Brown

**Affiliations:** Norwegian Radiation and Nuclear Safety Authority, P.O. box 55, Østerås, 1332 Norway

**Keywords:** HBRA, NOR and REE cereal uptake, Concentration ratio, Deposition, Radium, Thorium, Uranium

## Abstract

Some areas of the world have high natural background levels of naturally occurring radionuclides (NOR) and/or rare earth elements (REE). With high background radiation risk, knowledge about, for example, uptake by crops is required in the process of assessing radiation doses to humans. In the Fen complex, in Norway, underlying bedrock has elevated levels of both NOR and REE. In Mining hill where these bedrocks surface and there are also legacy mines, soils also have high background levels. Most of Fen complex is, however, covered by thick Holocene deposits but some of its agricultural soils are situated near the areas of surfacing carbonatite bedrock and legacy mines. We assess whether there has been any influence on the levels in the agricultural soils within the Fen complex from the areas with high NOR and REE soil background. We also determine uptake from soils by cereals into grain or grain with hull, and present concentration ratios (CR) for NOR and REE in this high background area. In particular, the REE CRs are an important augmentation to existing sparse datasets. We furthermore assess whether uptake can be biased by any soil particles adhering to cereal grain or grain with hull. We investigate whether washing (or not washing) and use of different acids for dissolution may affect ICP-MS results on common elements. We also address soil mass in cereal samples using Scandium and Titanium as tracers for soil particles and assess the potential magnitude of bias CR.

## Introduction

High background radiation areas (HBRA) involve higher risks (Sohrabi 2013; Aliyu and Ramli 2015). To assess radiological risk from naturally occurring radionuclides (NOR) in consumed foods, knowledge is needed about their uptake by crops from soils (UNSCEAR, 2008; IAEA 1990; IAEA, 2014, Pulhani et al. 2005; Renaud et al. 2015a). Such data from HBRAs are however few (Linsalata et al. 1991; IAEA 1990; IAEA, 2014). For rare earth elements (REE), there is some knowledge on human exposure (Brouziotis et al. 2022; González and Domingo 2024), bioavailability (Pagano et al. 2015; Fang et al. 2007; Guo et al. 2013), food content, and risks (Chen 2005; Chen and Zhu 2008; González and Domingo 2024; Gwenzi et al. 2018; Zaichick et al. 2011; Zhao et al. 2023).

Plant uptake of radionuclides (and elements) from soil can be described by a concentration ratio (CR), defined as the ratio between activity (concentration) of dry weight plant and soil (IAEA, 2014). If soil dust on the crop surface is not accounted for, estimated uptake may be biased. For Ra-226, such bias on CR may be 20-fold (IAEA 1990). In risk assessment models, deviations from default CR values may have large impacts on results, expressed, for example, as ingestion doses. Strategies to assess such bias are: to wash the biological sample under assessment (Renaud et al. 2015a; Azeez et al. 2019; IAEA 1990), to be sure the acid solution applied dissolves the biological sample and any soil particles adhering to plant surfaces separately before inductively coupled plasma mass spectrometry (ICP-MS), and to estimate the soil mass in plant samples using tracers for soil with low root uptake, like Scandium (Sc) and Titanium (Ti) (Cook et al. 2009; Hinton et al. 1995).

**Fig. 1:**
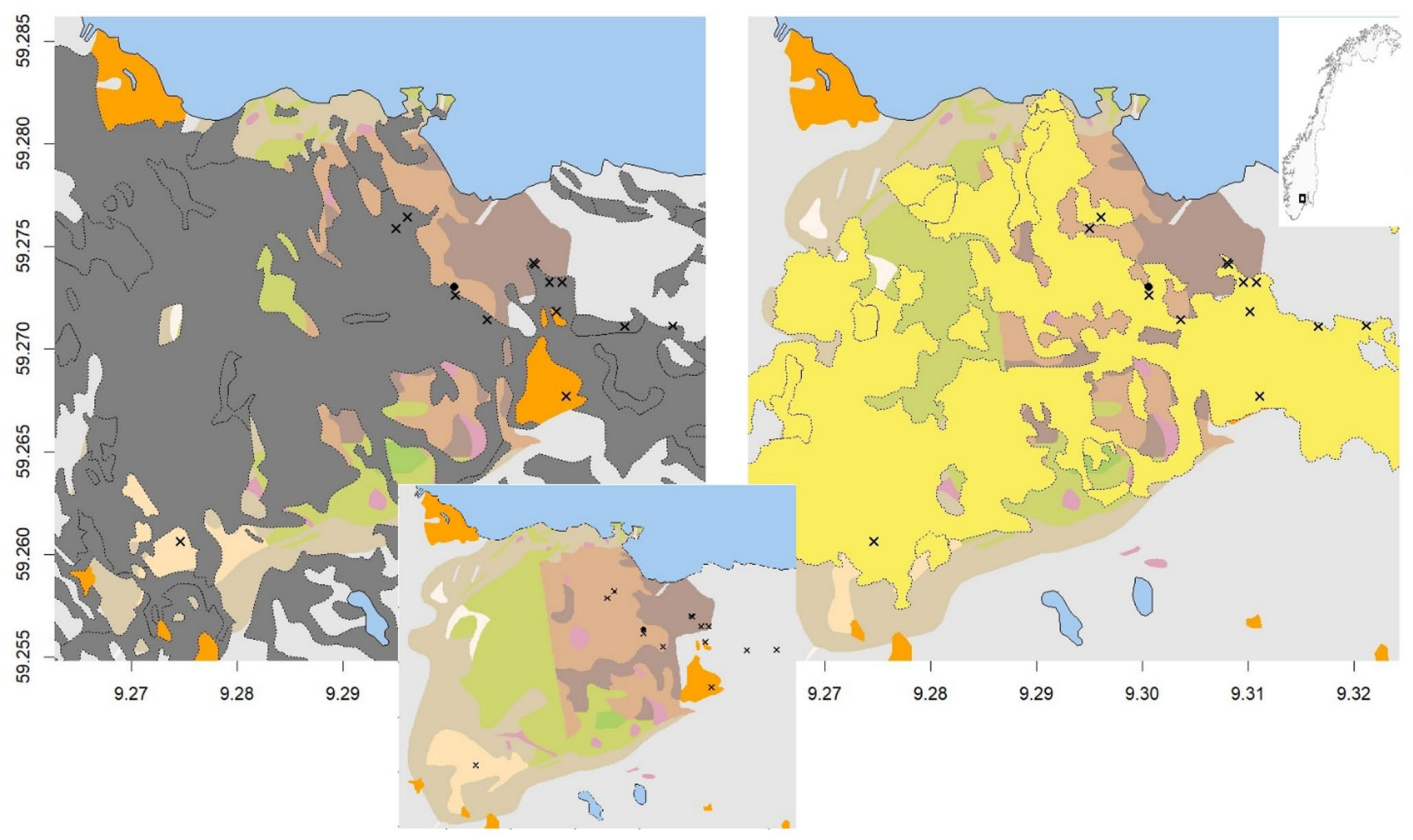
Maps of (1) Fen complex bedrock (middle), (2) Holocene deposits (grey layer), surfacing bedrock and bogs (orange) (left), (3) agricultural areas (yellow layer) (right). For bedrock, dark tan is redrock, light tan is FDC (aka ankerite), light green is søvite, green is vipetotitt, mauve is damtjernite, peach is melteigitt, beige is fenite, light peach is other carbonatites, and light grey is non-carbonatite bedrock (from Geological Survey of Norway and Norwegian Mapping Authority (https://kartkatalog.geonorge.no). X mark sampling locations. Axes are decimal degrees

We analyse agricultural soils and cereal grain or grain with hull (sheaf part) in an area with high levels of NOR and REE, the Fen igneous complex in Norway. In Fen complex, the Th-232 decay series is elevated in ferro dolomite carbonatite (FDC) and especially in redrock (Stranden 1985), while REE is elevated in several carbonatite bedrock and especially FDC (Dahlgren 2019). Adjacent to the study area, the “Mining Hill” has surfacing bedrock, legacy mines, and soils with high levels of NOR and REE (Popic et al. 2011, 2012b, 2014, 2020; Haanes and Gjelsvik 2021; Jakab et al., Submitted). Here, iron was mined for four centuries. However, the study area and most of the Fen complex is covered by Holocene deposits originating from other bedrock (Fig. 1). Winds and water erode, resuspend and transport soil particles (Errahmani et al. 2025; Whicker et al. 2021), and former legacy mining activities may also have involved transport of debris. We have therefore assessed agricultural soil and cereal from one field adjacent to theMining Hill, as well as cereals and soils from other agricultural fields in the Fen complex. Our goal was to assess the content of radionuclides and REE in agricultural soils and cereal grain or grain with hull, and to determine whether soil particles could bias estimates of uptake and the concentration ratios of NOR and REE.

## Methods

### Sampling and washing treatment of cereal

Samples were taken in both 2023 and 2024. In autumn 2023, one aggregate sample of wheat grain with hull (sheaf part) and three separate soils samples were taken from a field (59.27435 N, 9.30794 E) situated adjacent to the south side of the Mining Hill and the largest legacy mine (59.274169 N, 9.306781 E). In autumn 2024, aggregate samples of soil and cereal grain or grain with hull, respectively, were taken from ten additional fields in the Fen complex (Fig. 1). Aggregated soil samples consisted of two to four subsamples, depending on the size of the field. One aggregated soil sample was also taken in 2024 from the field sampled in 2023. In addition, one single soil sample was taken next to a field on the other side of the Mining Hill (south-western side, henceforth referred to as ‘EDGE’ and shown as a filled circle in Fig. 1), not representing agricultural soils but included due to its different red colour and potentially being weathered soil. Separate single soil samples were, in 2024, also taken from two of the fields where aggregated soil samples were taken. For all tables, box plots and analyses, it is the 2024 soil samples that are used (aggregated and single samples). The sample type of cereal harvested from each of the ten fields were: two of wheat grain, two of wheat grain with hull, three of oat grain with hull and three of barley grain with hull. A sample from 2023 of oat grain with hull was also included.

For each cereal grain or grain with hull sample, approximately 1 kg was taken (to have enough material for different treatments). Due to mixing in the thresher, the bulk sample was considered to represent the field. The threshing process mixes cereal both during threshing and when cereal is fed into the thresher container, but in this process any airborne dust of soil from the field might be mixed in with the cereal. In practice, this means that it is not possible to differentiate between adhered particles that originate from deposition (and soil resuspension) processes within the field prior to threshing and adhered particles that have been mixed in during the threshing process itself. Each cereal sample was handled as little as possible to minimise loss of any soil adhered to the plant/cereal surface. Cereal moisture is minimised by awaiting harvest (Reykdal et al. 2018) but nonetheless varies greatly (Johnson 1959). Therefore, we only assess dry matter levels of NOR and REE, for CR as well. The cereal samples were dried at 60 °C for six days in the same container. Soil samples were dried at 105 °C for 96 h, homogenised and sieved (2 mm), and water content was recorded. For 10 cereal samples, a subsample was taken and washed in approximately 5 L distilled water in a plastic tray by shaking in a sieve for 2 min. The tray was washed and distilled water replaced for each sample. After washing, soil particles were observed at the bottom of the white tray of some samples, supporting the expectation that soil particles may be adhered to the plant/cereal surface. Washed cereals were subsequently dried.

### Gamma spectrometry

To assess the level of Ra-226, Ra-228 (NOR) and Cs-137, samples were analysed using gamma spectrometry at low background using high purity Germanium detectors, controlled against a traceable source and with relative efficiencies of 23 to 50%. Soil was measured in 140 mL geometries and cereal (not washed) in 0.55 L Marinelli geometries. Soil samples were vacuum sealed in aluminium foil for 21 days to ensure secular equilibrium for Ra-226 progeny. Contents were assessed with uncertainty-weighting among energy lines of Ra-228 through Ac-228 at 338, 911 and 969 keV, Ra-226 through Rn-222 progenies Pb-214 at 295 and 352 keV and Bi-214 at 609 keV. Cs-137 was assessed using the 661.65 keV energy line. HPGe minimum detectable activity (MDA) ranged from 0.25 to 2.1 Bq kg^− 1^ dw for soil and from 0.07 to 4.1 Bq kg^− 1^ dw for cereal. Uncertainties (percent of one SD) ranged from 3 to 6.25% for soil and from 3 to 40% for cereal.

### ICP-MS

The contents of each, in total, of 36 elements (27 in 2023) were assessed in soil and cereal samples through ICP-MS, including the light REE’s: cerium (Ce), lanthanum (La), neodymium (Nd), praseodymium (Pr), yttrium (Y), samarium (Sm), europium (Eu), gadolinium (Gd), and the heavy REE’s: terbium (Tb), dysprosium (Dy), holmium (Ho), erbium (Er), thulium (Tm) and ytterbium (Yb). Titanium (Ti) and Scandium (Sc) were also included in the ICP-MS analysis, due to their low root uptake, as tracers for any present soil mass. Some common elements were also included, as well as some potentially toxic elements (PTEs)(Pourret et al. 2021). Activity concentrations were calculated for Th and U, assuming one mg kg^− 1^ being equivalent to 12.3 Bq kg^− 1^ for U-238, and one mg kg^− 1^ being equivalent to 4.1 Bq kg^− 1^ for Th-232. This was done to assess secular equilibrium with their progeny Ra-226 and Ra-228. After dissolving samples with acid (below), diluted samples were analysed on an extended range high resolution Element XR HR-ICP-MS (thermofisher.com), using certified calibration solutions and reference materials. The estimated analytical uncertainty for the ICP-MS results on unwashed cereal dissolved with an optimised acid solution was 2–4% for most elements, but higher for a few with elevated concentrations (Na: 58%, Mg: 14%, Ca: 22%, S: 28%, K: 20%). The estimated analytical uncertainty for the remainder of ICP-MS results were 13–29% for cereal and 11–34% for soil analyses (excluding Na, Mg, Ca, S).

### Acid dissolution treatments of cereal

Prior to ICPMS analysis, each sample was dissolved, and for the cereal samples, different acid solutions were attempted. The sample of wheat grain with hull from 2023 was dissolved in 7mL HNO_3_ + 1mL H_2_O_2_ and the associated soil samples were dissolved in 4mL HNO_3_ + 3mL HF + 1mL H_2_O_2_, digested at 200 °C for 20 min and diluted with 50mL distilled water (IFE laboratory). Then, the washed and unwashed cereal samples from 2024 were dissolved using the same acids with similar solutions and microwave digestion (SE-SOP-0128 at ALS laboratory according to SS-EN 13805:2014). The acid solution used for the 2024 soil samples, by comparison, had a slightly different acid solution containing HCl rather than H_2_O_2_ and a heatblock was used (SE-SOP-0039 at ALS laboratory according to SS-EN 13656:2003). ICP-MS measurements of soil sampled in 2024 were above MDA for 30 to 31 elements, while measurements of cereal collected in 2024 dissolved with the acid solutions described above were above MDA for 12 to 31 elements (mean: 26). Finally, a different acid solution was used to dissolve unwashed cereal samples from 2024 in an attempt to optimise also dissolution of any adhering soil particles by using an acid solution of 7mL HNO_3_ + 1mL H_2_O_2_ + 0.5mL HF, with 45 min @ 200 °C of microwave-assisted digestion (IFE laboratory). The hydrofluoric acid (HF) was thus introduced to give a better dissolution of any soil particles adhered to the surface of the cereal. For these 2024 un-washed cereal samples, measurements were above MDA in 21 of 30 elements across locations, including 6 to 14 REE (mean: 10), as well as Ti for all locations, Sc for all except two but U only in two locations. We used the optimised acid solution for cereal to assess results on elements not taken up over plant roots to assess the amount of soil present in cereal samples.

### Estimation of soil mass adhered to cereal

To further assess whether soil particles attach to cereal samples, the presence of elements in cereal, which are not known to be taken up over the root, was addressed. In particular, Ti and Sc have near zero root uptake and any amounts associated with plants can be used to estimate residual soil (Hinton et al. 1995; Cook et al. 2009). From the part of soil mass (mg kg^− 1^ dw) that such an element constitutes, the mass of soil (kg dw) present in a plant sample can be estimated from the concentration of the element in the plant sample. For example, for 1 kg of cereal, a Sc concentration of 20 mg kg^− 1^ dw would, therefore, with a soil concentration of 200 mg kg^− 1^ dw, indicate that 1/10 of a kg dw soil was present. Any small amounts of root uptake would bias such an estimate, but with the presence of substantial amounts of elements having near zero root uptake, the bias should be negligible. Any identified amount of soil in cereal samples was accounted for before calculating unbiased CR values for NOR and REE in cereal from the Fen complex.

### Soil parameter analyses

The soil samples from the field, sampled in 2023, were only analysed for pH, adding to soil (10 mL) distilled water (20 mL, 20 °C), shaking and measuring after 30 and 32 min using a Hach Sension + 374 multimeter and Supelco buffer solutions (pH 4.00, 7.00, 10.00) for calibration (IFE lab). The ten soil samples taken from fields in 2024 were analysed for the following parameters after drying (Eurofins labs https://www.eurofins-agro.com/no/jord): (1) percent dry matter, (2) soil type, identifying among the samples: silty medium sized sand (type 5), sandy silt (type 7), light clay (type 9), silty light clay (type 10) and organic soil (type 14), (3) classification according to percent clay, (4) total amount of organic carbon (TOC, percent per kg dw), (5) organic content by loss on ignition (OC, percent pr kg dw), (6) pH, (7) amount available for plant uptake (mg/100 g) of phosphorous (P-AL), potassium (K-AL), magnesium (Mg-AL), calcium (Ca-AL), sodium (Na-AL), (8) amounts of nitrate (NO₃⁻), ammonium (NH₄⁺) and total nitrogen (Kjeldal method), and (9) the ratio carbon to nitrogen (C/N). In addition, for the three fields closest to the Mining hill (GÅ1, GÅ2 and GÅ3), a soil sample (100 g) was sieved to identify the parts (grams of the 100 grams) of soil grain being clay (< 0.002 mm), fine silt (0.002–0.006 mm), medium silt (0.006–0.02 mm), coarse silt (0.02–0.06 mm), fine sand (0.06–0.2 mm), medium sand (0.2–0.6 mm) or coarse sand (0.6–2.0 mm). Furthermore, the mass of soil > 2.0 mm is given in grams.

### Statistics, data treatment and map

To compare the ICP-MS results from the different cereal treatments, we assessed the difference between each pair of treatments (unwashed vs. washed, and cereal acid dissolution vs. optimised dissolution). Since contents vary among elements by several orders of magnitude, the difference between the two treatment results per element, was assessed as the percentage of the untreated measurement. Whether differences across all elements were significant was tested with a one-sided Wilcoxon signed rank test (paired), which uses rank rather than absolute values. Measurements below the limit of detection (LOD) differed across elements and locations and among treatments. In cases where the compared treatments (one element at one location) had a LOD in one case and a measurement value in the other, the LOD was included for the comparison to increase statistical power. Under a null hypothesis of equal values, the use of LOD is conservative, since the measurement value is lower, and thus no bias will be introduced (except toward the null hypothesis).

Due to the small sample sizes and poor statistical power, no advanced statistical analyses were performed. Due to skewed distributions, Spearman rank correlations were performed between soil REE and NOR, and between REE and each PTE, as well as between either REE or NOR and each of the soil parameters. This was also performed for the CR of REE and NOR with soil parameters, and any effect on CR of cereal type was tested with a Kruskal-Wallis rank sum test.

All analyses and maps were made in R (R Core Team, 2016) using package SF (Pebesma and Bivand 2023) and map data on bedrock and Holocene loose mass deposition spatial distributions provided by the Geological Survey of Norway (NGU) and basic map data that was all downloaded from the Norwegian Mapping Authority web pages Geonorge (https://kartkatalog.geonorge.no).

## Results

### NOR, REE and PTEs in agricultural soils of Fen complex

**Fig. 2:**
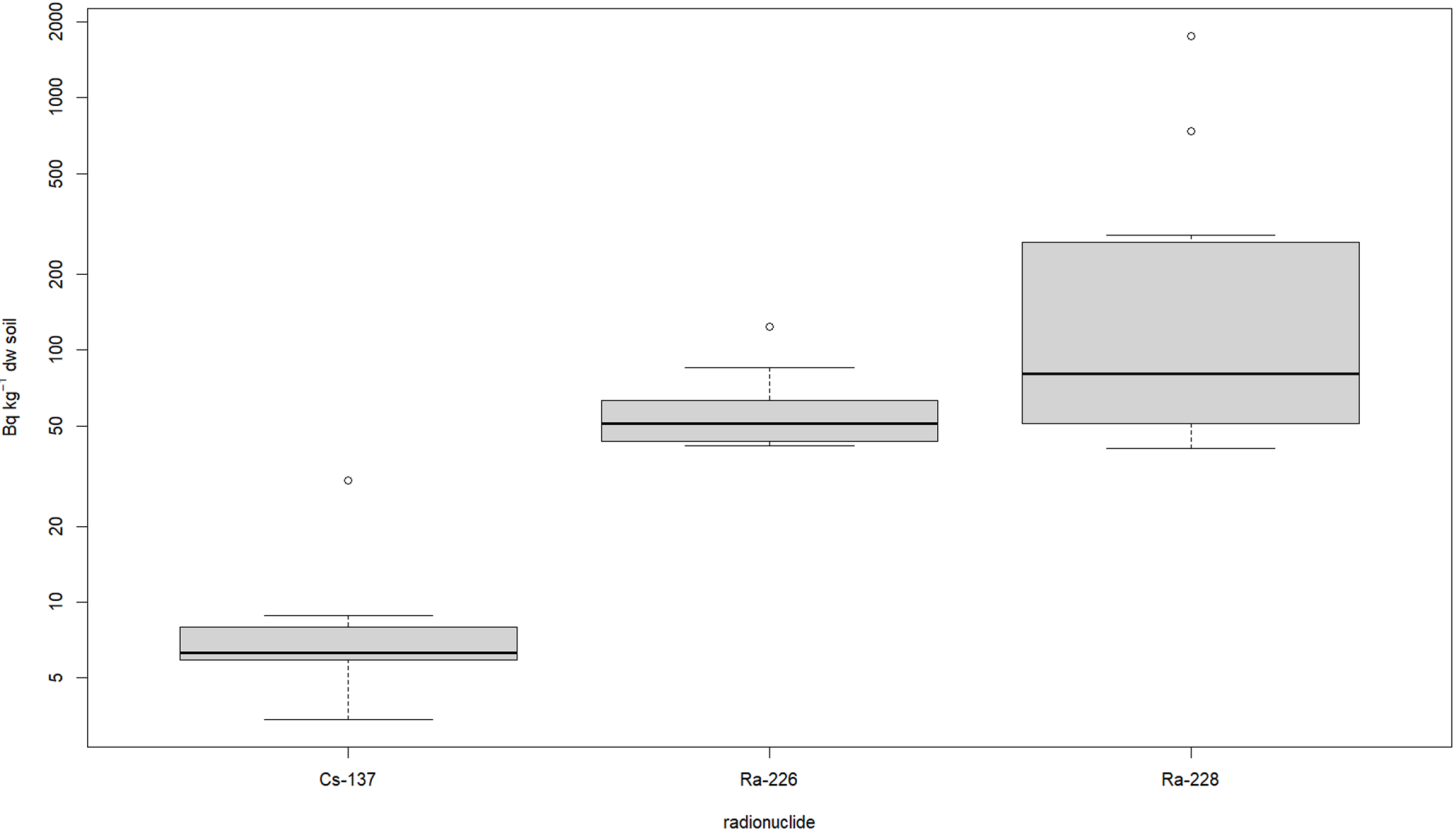
Box plot of content of radionuclides in aggregated samples of agricultural soils (dw) from Fen complex

NOR and REE had skewed distributions in the agricultural soils of Fen complex, particularly Ra-228 and some REE, with maximum values five to ten times as high as the median value. Levels of Ra-228 activity concentrations ranged from around 80 Bq kg^− 1^ (median) to outliers at 1800 and 730 Bq kg^− 1^ in the aggregated sample from the field closest to the Mining Hill and the largest legacy mine and in the sample taken next to a field (EDGE, not agricultural soil), respectively. By comparison, the agricultural field next to the latter (EDGE) had a value closer to the median among samples (120 Bq kg^− 1^). Within the field next to the Mining Hill, the single samples from 2023 in Ra-228 ranged from 600 to 1800 Bq kg^− 1^ (mean: 1300, median: 1600, SD: 640), illustrating a large heterogeneity of NOR in this field. Compared to Ra-228, Ra-226 had much less variation and lower levels, as did also Cs-137 (Table 1; Fig. 2). Details with data from separate locations will be sent upon request and can be found in Mendeley data repository (https://data.mendeley.com/datasets/sxvsf57jhf/110.17632/nrkdfkw942.1). Levels of Cs-137 were as elsewhere in the region.

**Table 1:** Range (min-max), mean, median and standard deviation (SD) of activity concentrations in aggregated samples (*n* = 11) from agricultural soils (Bq kg^− 1^ dw), within the Fen complex

Radionuclide	Min	Max	Mean	Median	SD
Ra-226	42	120	59	51	23
Ra-228	41	1800	270	80	470
Cs-137	3.4	31	8.0	6.3	6.6

The contents of progenitors U and Th of the assessed NOR decay series were also elevated in soil samples (Table 2). For Th, there were two outliers, the field adjacent to the Mining Hill and the largest legacy mine (450 mg kg^− 1^) and the EDGE sample taken next to a field (210 mg kg^− 1^). Excluding the outliers, the range of soil samples was 9.5 to 66 mg kg^− 1^. By comparison, the three 2023 samples from the field adjacent to the Mining Hill in Th ranged 47 to 150 mg kg^− 1^ (mean: 96, median: 88, SD: 53), demonstrating again a high heterogeneity of NOR in this field. Among soil samples (including the 2023 samples), there was secular equilibrium in soil between Th (ICPMS) and Ra-228 (HPGe). For U, there were two outliers in fields in the middle of the Fen complex, situated in or near Fen bog, Fnbg1 (77 mg kg^− 1^) and Fnbg2 (35 mg kg^− 1^), and excluding these the range was 3.3–10 mg kg^− 1^. For this decay chain there was also secular equilibrium, except for the two outliers where U levels were 17-fold and 8-fold of the median. Th was strongly correlated with the sum of REE (rho = 0.82, *p* < 0.001), and to some extent to the sum of HREE (rho = 0.38, *p* < 0.001), whereas U only was somewhat correlated to the sum of HREE (rho = 0.37, *p* < 0.001).

Among the 10 aggregate soil samples taken in 2024 from Fen agricultural soils and the EDGE sample, the sum of REE ranged from 250 to 3800 mg kg^− 1^ (mean: 840, median: 450, SD: 1000). The field closest to the Mining Hill and the largest legacy mine and the EDGE sample taken next to a field represent outliers of 2800 and 3800 mg kg^− 1^, respectively. For the soil samples taken in 2023 within the field adjacent to the Mining Hill, the sum of REE ranged 510 to 1600 (mean: 1500, median: 1100, SD: 840). Overall, REE comprised mostly light REE, and in particular Ce, Nd and La (Table 2). The highest values of Ce (1000 and 1700 mg kg^− 1^), Nd (640 and 700 mg kg^− 1^) and La (560 and 960 mg kg^− 1^) were found in the aggregated sample from the field adjacent to the Mining Hill and the largest legacy mine and in the EDGE sample taken next to a field. The field adjacent to the latter EDGE sample had 1/9 to 1/7 as high values of Nd, Ce and La. Ranges in the rest of Fen agricultural soils were for Ce 85 to 340 mg kg^− 1^, for Nd 41 to 240 mg kg^− 1^ and for La 46 to 170 mg kg^− 1^. Heavy REE had, as expected, much lower concentrations than LREE with a ratio of around 1/20 (Table 2), with the two highest sums of HREE in the field closest to the Mining Hill and the largest legacy mine and in the EDGE sample taken next to a field, with 49 and 40 mg kg^− 1^, respectively.

**Table 2:** Range (min-max), mean, median and standard deviation (SD) of REE and NOR contents of aggregated samples of agricultural soils (mg kg^− 1^), as well as Sc and Ti, within the Fen complex

REE/NOR/Ti	Minimum	Maximum	Mean	Median	SD
Y	23	110	51	45	22
**La**	**46**	**960**	**180**	**84**	**260**
**Ce**	**85**	**1700**	**340**	**160**	**470**
Pr	11	170	39	21	52
Nd	41	740	170	81	240
Sm	6.8	99	24	15	29
Eu	1.6	20	5.5	3.2	6.3
Gd	6.1	47	16	11	13
**Sum LREE**	***240***	***3900***	***820***	***430***	***1000***
Tb	0.79	4.9	2.0	1.5	1.3
Dy	4.0	23	11	8.7	5.3
Ho	NA	NA	NA	NA	NA
Er	2.3	11	5.4	5.0	2.0
Tm	NA	NA	NA	NA	NA
Yb	3.2	9.8	4.9	4.5	1.7
**Sum HREE**	***10***	***49***	***23***	***19***	***9.8***
Th	9.5	450	69	22	120
U	3.3	77	12	4.4	20
Sc	2.9	31	11	9.4	7.4
Ti	1300	14,000	5100	4300	2900

Among common elements in Fen agricultural soils, some Fe values stood out as high among samples (Table 3). Some Fen bedrock are hematized (Dahlgren 2019), and input from these may explain this. The highest Fe levels were found in the field adjacent to the Mining Hill and the large legacy mine where iron-rich redrock is found, but the two second highest values were found on the southern side of the Fen complex, in an area with surfacing melteigite (Fig. 1) and in the EDGE sample taken next to a field. For the latter, the adjacent sampled field (next to EDGE) had a Fe value around half as large, suggesting less input from the Mining Hill. High but variable Ca values gave support to this, as can be expected with input from carbonatite bedrock. Regarding the levels of Nb in the carbonatites, the observed soil levels of Nb are also as expected. Positive correlation with the sum of REE was found for Fe (rho = 0.56, *p* < 0.001), Zn (rho = 0.67, *p* < 0.001) and Nb (rho = 0.63, *p* < 0.001), but correlations were even stronger for the sum of HREE and either Fe (rho = 0.94, *p* < 0.001) or Zn (rho = 0.75, *p* < 0.001). Among the PTEs, the highest and outlier values of Zn and Cd were found in the EDGE sample taken next to a field and the second highest value in the field adjacent to the Mining Hill and the third highest (the last above 200 mg kg^− 1^) in the most southern sampled field of the Fen complex. This field also had the highest and outlier value of Cr.

**Table 3:** Range (min-max), mean, median and standard deviation (SD) of some common elements and heavy metals contents in agricultural soils (mg kg^− 1^), within the Fen complex

Element	Minimum	Maximum	Mean	Median	SD
Fe	12,000	97,000	43,000	33,000	24,000
Ca	8800	83,000	21,000	12,000	21,000
Mg	3100	19,000	7700	6800	4100
Na	3900	16,000	14,000	14,000	3100
P	900	6500	2300	1400	1700
S	190	5900	870	390	1500
Zr	87	670	310	290	120
Nb	16	560	120	56	150
Zn	65	530	140	95	120
Cr	50	220	85	73	44
Cu	8.5	66	19	14	15
Pb	20	61	31	28	11
Li	3.2	31	18	18	6.9
As	3.1	6.9	4.6	4.4	1.1
Cd	0.06	1.1	0.30	0.18	0.27

For Se, all measurements were all below MDA (2 mg kg^− 1^ dw)

### Soil properties of agricultural soils from Fen complex

All soil samples had pH values from around 5 to 6.5 (Table 4), as may be expected with any weathered carbonatite bedrock with CaCO_3_ acting as an acid buffer. This is supported by the content of Ca available to plants (Ca-AL) among soil samples. The content of available sodium (Na-AL) was low in all samples (< 2 mg/100 g dw). There was little variation in phosphorus (P-AL), potassium (K-AL) and magnesium (mg-AL) among the samples (Table 4). The percentage of clay was either medium (10–25% dw), low (5–10% dw), or in one instance very low (< 5% dw). The latter sample (Fnbg1) stood out as an organic soil with outlier values also on higher TOC and loss on ignition, water content, calcium, ammonium, nitrate and total nitrogen (see maximum value Table 4), but being in the lower end for all NOR (Table 1) and REE (Table 2). The other soil samples had total organic carbon (TOC) percentage from 1 to 6 (dw), nitrate (NO₃⁻) from 4 to 9 (mg/100 g dw), ammonium (NH₄⁺,) 0 to 7 (mg/100 g dw), and total nitrogen (Kjeldal method) up to 0.7% (dw). Even so, the C/N did not vary much among soils. Among these soil parameters no correlations were found with REE or Th, but U (and Ra-226) was correlated with loss on ignition (rho = 0.58, *p* < 0.001) and TOC (rho = 0.56, *p* < 0.001).

The soil grain size in the three fields adjacent to the Mining Hill and the largest legacy mine, was dominated by high proportions of coarse and medium silt (20 to 30 g per 100 g dw), as expected, but the closer field had twelve times more coarse sand and more than twice as much (by mass) of particles with grainsizes > 2 mm. These coarser sizes may originate from legacy mining activity debris. The higher levels of Th and Ra-228 in this field and proximity to the large mine opening may suggest mixing of legacy waste into the field.

**Table 4:** Range (min-max), mean, median and standard deviation (SD) of soil properties (Param) of agricultural soil samples (*n* = 10) from 2024 in Fen complex, including denominations

Param	Minimum	Maximum	Mean	Median	SD
% dw	31	79	69	74	14
pH	5.2	6.6	6.0	6.2	0.50
mould, % dw	2.6	76	11	4.5	21
P-AL, mg/100 g	5.0	14	9.2	9.0	3.0
K-AL, mg/100 g	9.0	25	15	14	5.1
Mg-AL, mg/100 g	2.0	28	14.7	12	7.9
Ca-AL, mg/100 g	33	420	160	130	110
OC, % dw	3.6	76	13	6.2	21
TOC, % dw	1.3	27	5.0	2.7	7.7
NO₃⁻, mg/100 g	3.9	40	9.1	6.5	10
NH₄⁺, mg/100 g	0.00	19	4.5	3.2	5.2
N Kjeldal method, % dw	0.23	3.0	0.61	0.37	0.80
C/N	6	9	7.8	8.0	0.98

### Effects of cereal treatments and estimate of soil mass on cereal samples

**Fig. 4:**
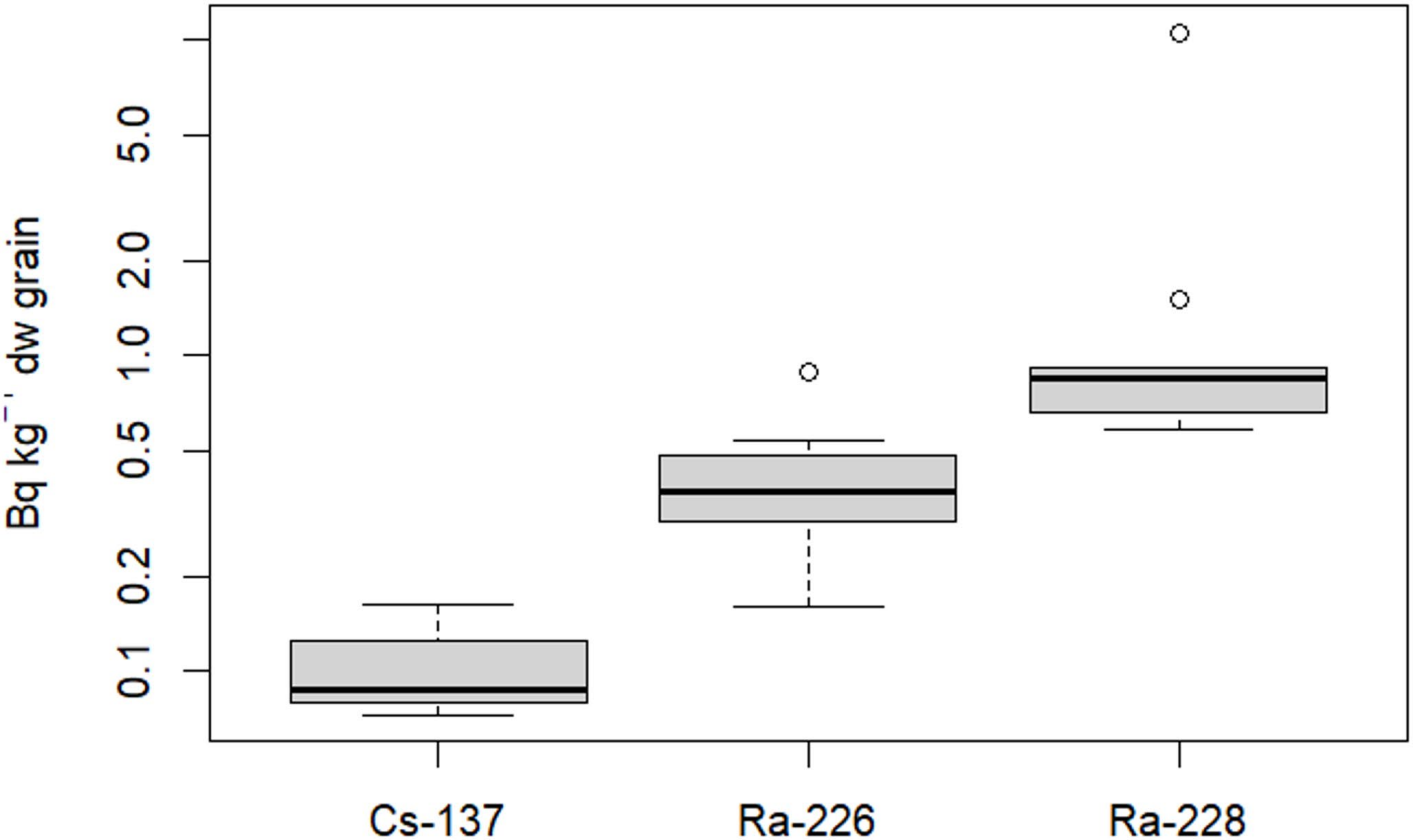
Levels of Ra-226, Ra-228 (NOR) and Cs-137 in cereal from Fen complex

Soil particles were observed at the bottom of the white plastic tray after washing of some of the cereal samples. Around half of the ICPMS results on un-washed and washed cereal (common biota digesting acid solution) were below limit of detection (LOD). Among pairs of measurements among locations and elements, the measurements from unwashed cereal seemed to be higher than for washed cereal, but the difference was not significant in a Wilcoxon signed rank test (V = 3975, *p* > 0.14), and this remained the case if LOD values were included (*p* > 0.21). By comparison, a quarter of the ICPMS cereal results using an optimised acid solution were below LOD. Compared to the ICPMS results on cereal dissolved with a common acid solution, the optimal acid solution had significantly higher measurement values in a Wilcoxon signed rank test (V = 3723, *p* < 0.023, Fig. 4). We include here, however, also the results from the common acid solution on Th since for the optimal acid solution, ICPMS results were consistently below LOD (0.018 mg kg^− 1^). A lower LOD in the ICPMS for the common acid solution involved three measurement values for Th, 0.003, 0.003 and 0.005 mg kg^− 1^ (second and third closest field to largest legacy mine and Fen bog).

Finally, assuming no root uptake for Sc and Ti, the presence of these elements in ICPMS of cereal samples suggested ranges of 1.1 to 3.7 mg soil per gram cereal (mean: 1.6, median: 1.2, SD: 0.87) for Sc and 0.039 to 1.7 mg soil per gram cereal (mean: 0.27, median: 0.11, SD: 0.49) for Ti. For Ti, the maximum value was an outlier from the field adjacent to the Mining Hill and the largest legacy mine and the second largest estimated soil mass was 0.28 mg per gram plant. This locality gave a value that was below LOD for Sc (2023 cereal ICPMS) but the LOD value was used for estimating soil mass to provide a conservative estimate of the bias from soil mass, of 1.6 mg soil per gram plant, equal to the mean value. For each locality, the concentration ratio from soil to plant was then calculated. The percentage of the bias on CR that the estimated soil mass (Sc and Ti calculated) would constitute was also calculated, involving some variation for Ti and large variation for Sc estimates (Tables 5, 6 and 7).

### NOR, REE and PTE of cereal from Fen complex

In cereal, the highest level of Ra-228 (Th-232 decay chain) was in the sample from the field adjacent to the Mining Hill and the largest legacy mine, which was tenfold compared to the other cereal samples (Table 5; Fig. 4). The second highest Ra-228 cereal value was from the next and second closest field to the largest legacy mine (1.5 Bq kg^− 1^ dw). Excluding these two samples, the other Ra-228 measurements were lower than 1 Bq kg^− 1^. CR for Ra-228 ranged from 0.0030 to 0.014, with the two maximum values at 0.011 and 0.014 situated near the Fen bog (FnbgA and FnbgB). Levels of Ra-226 in cereal were even lower but with an outlier at 0.89 Bq kg^− 1^. Cs-137 was also low (Table 5). CR for Ra-226 ranged from 0.0037 to 0.019, with the two maximum values at 0.013 and 0.019 in fields at some distance from the Mining hill (FenSchool1 and FenSchool2). For ICPMS results, LOD for Th and U in cereal were 0.17 and 0.002 mg kg^− 1^, respectively, and there was only one Th measurement and three U measurements in cereal above LOD. The 0.52 mg kg^− 1^ measurement of Th in cereal translates to 2 Bq kg^− 1^ cereal dry weight. It was from the field adjacent to the Mining Hill with the highest soil level, suggesting a higher uptake of Ra-228. The three U measurements above LOD translate to from 0.0037 to 0.64 Bq kg^− 1^ cereal dry weight. This suggests a higher uptake for two of the fields, with tenfold (locality Fnbg2 ) and hundred-fold surplus of Ra-226 (locality FnbgB), the former in the Fen bog. These three CR values of U involved one high (CR = 0.012) and two low. The only statistically significant correlation of radium isotope CR with soil parameters was CR of Ra-228 with available K in soil (rho = 0.72, *p* > 0.05).

**Table 5:** Range (min-max), mean, median and standard deviation (SD) of activity concentrations measured in cereal samples (Bq kg^− 1^ dw) from the Fen complex, and estimates of CR before and after accounting for soil particles using Ti and Sc as tracers, respectively

Radionuclide	Min	Max	Mean	Median	SD	CR_biased_	% CR_Ti_	% CR_Sc_
Ra-226	0.16	0.89	0.43	0.37	0.24	0.0086	5.5	21
Ra-228	0.58	11	1.9	0.85	3.3	0.0077	4.8	20
Cs-137	0.07	0.16	1.11	0.09	0.05	0.012	6.6	57

**Fig. 5:**
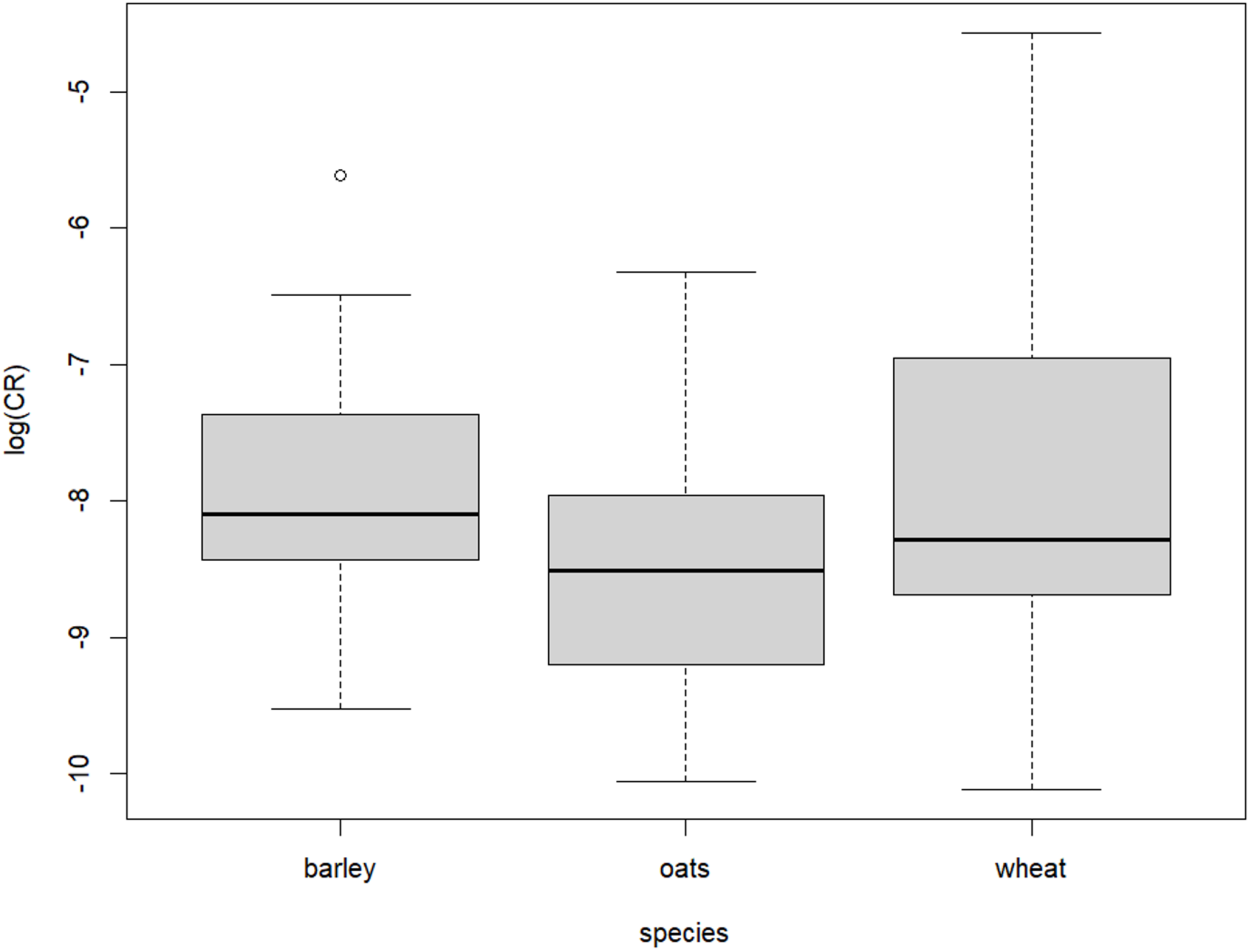
CR (log scale) values identified for REE among the three assessed cereal types

**Fig. 6:**
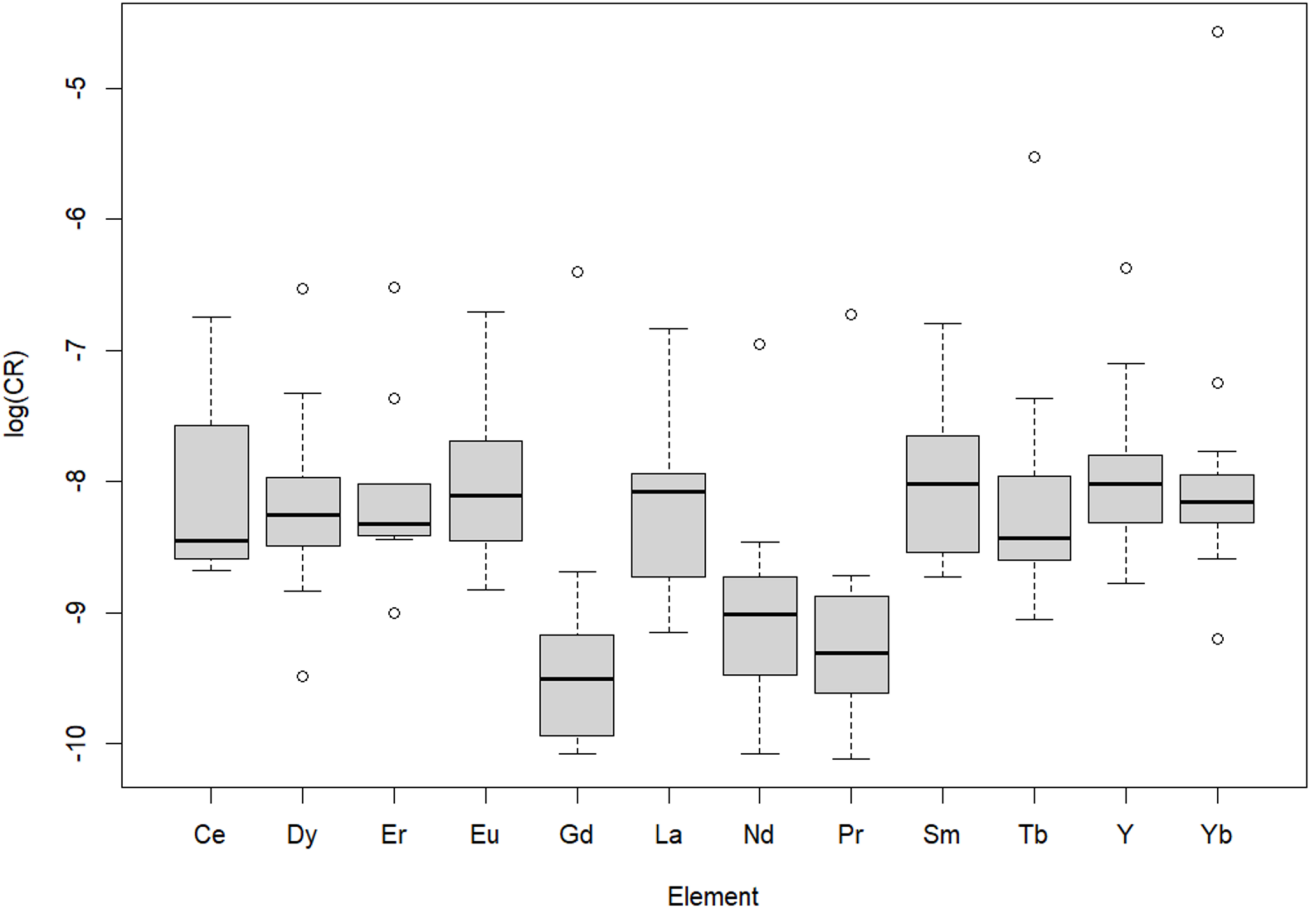
CR (log scale) among the REE elements assessed

The cereal content of REE (Table 6) was skewed and variable, with the sum of REE ranging from 0.013 to 3.3 mg kg^− 1^ (mean: 0.32, median: 0.042, SD: 0.89), with outliers of 3.3, 0.11 and 0.11 mg kg^− 1^ from the field adjacent to the Mining Hill and the largest legacy mine, the field next to that and from the Fen bog field, respectively. The latter was also the one with the largest deviation in Ra-226 secular equilibrium. Most REE in cereal samples consisted of LREE, and especially Ce, as well as Nd and La (Table 6). The HREE to LREE ratio in cereal was from 0.04 to 0.15. The estimated uptake and CR (the dry weight ratio to soil content) of REE elements (excluding Sc) in unwashed cereal ranged from 0.000041 to 0.010 (mean: 0.00054, median: 0.00025, SD: 0.0011) when the field adjacent to the Mining hill and the legacy mine was included but when excluded with a maximum of 0.00083 (mean: 0.00025, median: 0.00022, SD: 0.00017). Gd, Nd and Pr seem to have a slightly lower CR (Fig. 6). Within the field adjacent to the Mining hill and the legacy mines, REE CR ranged from 0.00096 to 0.010, demonstrating a high degree of heterogeneity. Across all samples, no soil parameters were significantly and positively correlated with any of the LREEs, but there was a significant difference in REE CR among the three assessed species of cereal (chi-square = 6.1, *p* < 0.05, df = 2, Fig. 5).

Cereal Sc had little variation and was lower than Ti, which had more variation (Table 6). Using the amount of soil mass estimated if no root uptake of Ti and Sc is assumed (see methods), the percent of bias in CR due to soil mass would range from 2 to 280% when estimated with Ti and from 16 to 4200% when estimated with Sc (see Table 6 for separate REE). For Ti estimates, 29 of 121 cases involve a bias from soil mass on CR of more than 100%, which suggest some root uptake, while for Sc estimates more than 100% bias is in 85 of 97 cases suggests an even higher root uptake. The one measurement of Th above LOD was from the field adjacent to the Mining Hill and the largest legacy mine, but due to the low mobility of Th and anticipated close to non-existent root uptake of Th, it may be due to soil mass (and a true bias on CR), even though the estimate higher than 100% bias indicates some root uptake. For U, the CR values on the other hand are as expected with mobility and root uptake, as also suggested by the much higher than 100% estimated bias from soil mass on CR. For the other NOR, the smaller differences in activity concentration between soil and plants (Tables 1 and 5) involves smaller estimated biases from soil mass.

**Table 6:** Range (min-max), mean, median and standard deviation (SD) of REE contents (mg kg^− 1^) in Fen complex cereal, and estimates (mean among localities) of CR, including estimated percent bias from soil particles using Ti and Sc as tracers (mean among localities)

Element	Min	Max	Mean	Median	SD	CR_biased_	% CR_Ti_	% CR_Sc_
Sc	0.010	0.027	0.013	0.012	0.0053	0.0016	7.3	
Y	0.0061	0.19	0.028	0.013	0.051	0.00047	41	560
**La**	**0.0066**	**0.61**	**0.090**	**0.015**	**0.21**	**0.00036**	**76**	690
**Ce**	**0.024**	**1.2**	**0.32**	**0.027**	**0.59**	**0.00044**	**89**	**260**
Pr	0.0008	0.18	0.024	0.0015	0.063	0.00023	140	**1800**
Nd	0.004	0.71	0.072	0.0077	0.21	0.00020	130	1500
Sm	0.0011	0.11	0.013	0.0037	0.031	0.00042	50	650
Eu	0.0005	0.025	0.0031	0.0009	0.0073	0.00040	50	590
Gd	0.0005	0.077	0.0077	0.0007	0.023	0.00024	150	2000
**∑LREE**	***0.010***	***3.10***	***0.30***	***0.038***	***0.85***			
Tb	0.0002	0.019	0.0023	0.0003	0.0059	0.00069	45	640
Dy	0.0008	0.034	0.0048	0.0020	0.0093	0.00039	51	780
Ho**	0.0004	0.0011	0.00064	0.0005	0.00028			
Er	0.0007	0.016	0.0026	0.0011	0.0045	0.00040	51	660
Tm*/**	0.0005							
Yb	0.0005	0.10	0.0096	0.0012	0.029	0.0012	37	670
**∑HREE**	***0.0017***	***0.17***	***0.019***	***0.0047***	***0.046***			
Th*	0.52					0.0012	148	140
U	0.0003	0.052	0.018	0.0018	0.029	0.0040	210	1200
Ti	0.20	7.1	1.0	0.45	1.9	0.00027	NA	1600
Lu*/**	0.0005							

*only one sample above MDA – one measured value

** no soil values

The levels of common elements in cereal were higher, as reflected in the CR values and from active uptake as expected (Tables 3 and 7), but with variation. The levels of PTEs were lower but with CR values one to two orders of magnitude higher than for REE, except for Pb (Table 7). Especially Zn and Cr had high cereal levels, but with outliers in CR only for Cr in the most southern locality and one in the Fen bog (FnbgB). The only correlations of PTE with soil parameters were for the CR of Pb and pH (rho = 0.83, *p* < 0.01), and the CR of Cu with available nitrogen (rho=-0.62, *p* < 0.05), TOC (rho = 0.77, *p* < 0.01) and loss on ignition (rho=-0.82, *p* < 0.01).

**Table 7:** Range (min-max), mean, median and standard deviation (SD) of some common elements and heavy metals in cereal (mg kg^− 1^), within the Fen complex, and estimates of CR, including estimated percent bias from soil particles using Ti and Sc as tracers (mean among localities)

Element	Min	Max	Mean	Median	SD	CR_biased_	% CR_Ti_	% CR_Sc_
Fe	32	180	53	43	40	0.0015	16	110
Ca	190	700	380	320	160	0.027	0.78	9.0
Mg	550	1400	1100	1200	260	0.19	0.27	1.0
Na	20	39	31	32	5.6	0.006	13	63
P	2700	4200	3400	3600	490	2.5	0.020	0.11
S	800	1700	1400	1400	250	3.7	0.019	0.25
Zr	NA							
Nb	NA							
Zn	**11**	**26**	**20**	**21**	**4.3**	0.22	0.059	0.78
Cr	**1.5**	**23**	**5.3**	**3.8**	**6.0**	0.072	0.20	2.9
Cu	2.5	4.3	3.7	3.4	0.72	**0.27**	0.086	1.8
Pb	0.02	0.03	0.024	0.02	0.0053	0.00089	15	240
Li	*							
As	0.11	0.19	0.15	0.15	0.025	**0.038**	0.35	3.4
Cd	*							

* only below LOD of 0.12 mg kg^− 1^ for Cd and 0.18 for Li

## Discussion

Only some of the soil samples in this study had significantly elevated NOR levels. The median and most common levels in the agricultural soils of the Fen complex were within and similar to the soil NOR averages of other countries, which ranged from 10 to 110 Bq kg^− 1^ dw for U-238, 4 to 126 Bq kg^− 1^ dw for Ra-226, and 10 to 95 Bq kg^− 1^ dw for Th-232 (UNSCEAR, 2008; IAEA, 2014). This suggested a dominance in the agricultural soils of the Fen complex of Holocene deposits, as expected. However, the NOR outliers, i.e., the two closest soil samples to the Mining hill and legacy mines, had twenty times higher values for Th and Ra-228, respectively, up to 1800 Bq kg^− 1^ dw for Ra-228. The next four closest fields had Ra-228 levels above 100 Bq kg^− 1^ dw. The same pattern applied for Th. For U, the two outliers were situated in the Fen bog ( Fnbg1 and Fnbg2) and did not have correspondingly high Ra-226 values, representing also outliers for secular equilibrium, as well as for ammonium, TOC and loss on ignition, but not pH. This could be related to redox potential and uranium mobility. For REE, Fe, Zn and Cd, the same two localities as for NOR represented outliers. This was as expected given the correlation between NOR and REE in the Fen complex (Jakab et al., Submitted). Compared to REE levels in most world soils (Chen et al. 2020; Ramos et al. 2016; Fang et al. 2007), the two outliers had tenfold concentrations of REE, mostly LREE (Table 3), while the other soil samples had two to threefold. Elevated levels of REE were also found in soils of US, Brazil, Cape Verde, Spain, UK, and Italy (Ramos et al. 2016). However, even though the two outliers in our study were 1/7 and 1/14 of those concentrations found at the REE mining site with the highest levels within China (Li et al. 2010; Wang and Liang 2015), they were in a range from similar to four to ten times the levels, in total REE, reported for other REE mining sites in China (Liu et al. 2019; Li et al. 2013).

It has been shown that soil within the Mining Hill has even much higher levels of NOR and REE (Haanes and Gjelsvik 2021; Popic et al. 2011, 2012a; Jakab et al., Submitted) compared to the levels we have reported in the present study. Both redrock in the Mining Hill, and FDC (see Fig. 1), had elevated levels of radionuclides from the Th-232 decay series and REE (Stranden 1985; Dahlgren 2019), and within the Mining Hill, soil REE can be over 4000 mg kg^− 1^ (Jakab et submitted). In the Mining Hill, there were no Holocene deposits and both weathering of surfacing bedrock and iron legacy mining, over a period of four centuries, probably contributed to soil formation. The two outliers among agricultural soils in the present study suggested a higher input from Fen bedrock than the other measured samples, probably in the form of soil or mining debris from the Mining Hill. This seems plausible since these sites were located closest to the Mining Hill and such dispersion may have operated for centuries, by wind, water and/or human activities, including the legacy mining. The outlier with the highest REE level was the EDGE soil sample taken next to a field on the south-west side of the Mining Hill, close to both FDC and the Mining Hill. Compared to this sample, FDC bedrock had up to two orders of magnitude higher levels of REE (Dahlgren 2019) and the close proximity to FDC may explain this outlier. By comparison to FDC bedrock, the bedrock levels at the largest REE mining site in China were double (Lai and Yang 2013) and contaminated soil levels were sevenfold (Li et al. 2010), suggesting a much higher input to soil by mining activities in China than in the present study. The other outlier, from the field adjacent to the Mining Hill and the largest legacy mine, was higher in NOR but second in REE in the present study, which might be expected, given the assumed input from redrock, which was significantly higher in NOR. By comparison, REE was enhanced in several Fen bedrocks (Dahlgren 2019) and the higher than global level of REE in most of the agricultural soil samples suggested a general input by weathering over geological time across the area. The Fe outlier in the most southern field may also be due to weathering, as hematized melteigite bedrock is exposed here, which is also suggested by the outlier value of Ca in this sample. For the field adjacent to the Mining Hill where the highest NOR values were found, in spite of ploughing, the variation in NOR activity was threefold. NOR distributions are often highly heterogenous in HBRAs (IAEA, 2014). An input by legacy mining was, however, further supported by the overrepresentation of coarser particle fractions (> 2 mm) within this field, which might be expected from loss of the smallest size fraction mine tailings during transport.

For cereal, the most significant, NOR outlier value was the 11 Bq kg^− 1^ dw Ra-228 measurement from the field adjacent to the Mining Hill and the largest legacy mine, where the corresponding value of Th-232 was 2 Bq kg^− 1^ dw. This points to a higher uptake of radium, as expected given the low mobility of Th, and an impact of legacy mining. The cereal from the second closest field had the second highest Ra-228 (1.5 Bq kg^− 1^ dw). For comparison, another high background area, Kerala in India, had radium levels in cereal (rice) reaching 0.78 Bq kg^− 1^ dw (IAEA 1990). For wheat grain, normal NOR ranges were from 0.03 to 0.10 Bq kg^− 1^ dw of Th-232, and 0.06 to 0.23 Bq kg^− 1^ dw of Ra-226 and Ra-228 (IAEA, 2014, Lindahl et al. 2011; Choi et al. 2008). Enhanced levels of NOR in wheat grain were also found elsewhere in India with up to 1.2 and 0.8 Bq kg^− 1^ dw of Th-232 and Ra-226 (Pulhani et al. 2005) but in France and Germany with up to 1.5 and 5.6 Bq kg^− 1^ fw of Ra-226 and Ra-228 (Renaud et al. 2015b).

For cereal REE, the field adjacent to the Mining hill and the largest legacy mine represented an outlier with a hundredfold of REE compared to the lowest values. The EDGE soil sample did not have any corresponding plant sample (as it was taken next to the field). Compared to the REE levels in cereals, including wheat, from areas where soil is contaminated from REE mining (Wang et al. 2020; Zhuang et al. 2017), the most significant outlier of the present study was one order of magnitude higher than their maximum recorded value while median values were similar. Similarly high REE levels have also been found in maize uptake experiments using mining soil and waste rock, with even two orders of magnitude higher levels in roots than shoots (Guo et al. 2013). Surprisingly, from two to five orders of magnitude higher levels of REE than in the Fen cereal outlier were found in wheat shoots grown in soil with less REE levels than Fen soil, even though this wheat was also rinsed with water (Fang et al. 2007). Clearly, variation in cereal REE uptake is large.

The enhanced Belgian and French NOR levels of cereal (above) were of cereal that was washed and peeled (Lindahl et al. 2011; Renaud et al. 2015b). However, in some cases, washing of above-ground vegetation has involved no differences in results (Tyler and Olsson 2001). In the present study, the washing of the cereal had a non-significant effect on ICPMS results. The presence of soil particles on the cereal became obvious, however, when using an optimised acid solution that dissolved both cereal and adhering soil particles. The ICPMS results from unwashed cereal samples using this optimised acid solution thus gave significantly higher measurements than the results from unwashed cereal dissolved with an acid solution commonly used to dissolve biological samples that does not fully dissolve soil. The presence of adhered soil particles was also strongly suggested by the Ti and Sc measured for cereal samples (optimised acid solution), since root uptake is low for these elements and adhering soil particles most likely explain their presence. It is known that the bias from deposited soil dust on plant surfaces can be large, as for Ra-226 where cereal CR can be up to 20 times higher than an unbiased CR of 0.06 (IAEA 1990). However, whether adhering soil particles originates from deposition processes (wet or dry) prior to threshing or whether they are introduced during threshing cannot be determined by the present study. Moreover, the method using Ti and Sc to estimate soil mass in the present study, did not give unambiguous results, probably due to uncertainties in the ICPMS measurements. This estimation method should probably not be used to interpret the exact amount of soil mass per cereal sample. For REE (Table 6), the estimated bias on CR thus seemed to be from plausible to high, for Ti, while extremely overestimated for Sc. For NOR activity concentrations (Table 5) on the other hand, the estimated bias on CR seemed plausible for Ti and somewhat high for Sc. The soil mass estimates can therefore not be viewed as exact but rather as support for the contention, in line with the optimised acid treatment, that cereal samples can have associated soil particles that should be accounted for when assessing risk from ingestion.

Without accounting for a bias from soil mass, the CR values of the cereal in the present study were mostly within normal ranges. Normally, for the Ra isotopes, cereal CRs have been shown to range from 0.00008 to 0.67 with a geometric mean of 0.017 (IAEA, 2014, Vandenhove et al. 2009), which is similar to the highest Fen values for cereal. Higher CR of Ra-226 were even found in India HBRA for wheat (Pulhani et al. 2005), as well as for grass in the Fen complex (Popic et al. 2020). Similar values were found for New Zealand crops (Pearson et al. 2016) and for both Ra isotopes in Belgian wheat (Lindahl et al. 2011). A possible explanation of the low CR radium values in some Fen agricultural soil samples may be found in the high Ca level and pH, as Ra is a chemical analogue to Ca. Also, two of the CR values of U were lower than reported for cereal in other regions (Pulhani et al. 2005; Vandenhove et al. 2009), as well as lower than for grass in the Fen complex (Popic et al. 2020), while the highest CR of U was similar. Notably, measurements of cereal and soil were on a much lower level for the two smaller CR values, involving higher measurement uncertainties, which involved a higher uncertainty also in these CR values. The CR of Th for Fen cereal was similar to values reported from elsewhere (IAEA, 2014, Vandenhove et al. 2009; Lindahl et al. 2011). However, considering the observed soil heterogeneity among the separate samples from this field, it was noteworthy that CR ranges only from 0.0035 to 0.012 for Th, 0.010–0.018 for U, 0.006–0.019 for Ra-228 and 0.006–0.008 for Ra-226.

For REE, the CR values from the present study make an important contribution to the scientific literature especially with regards to risk assessment. We found very few publications on crop and cereal CR, with one exception reporting relatively similar CR of REE values (Sheppard et al. 2010), except compared to the outliers from the field adjacent to the Mining Hill and the legacy mines. Here, variation spanned three orders of magnitude, probably reflecting heterogeneity, since the soil parameters of this locality were not different from the other agricultural fields. Factors known to affect the CR of REE include the degree of fertilisation and levels of nitrogen (Ramos et al. 2016), and since REE uptake is linked to Fe uptake (Brioschi et al. 2013), the outlier values and heterogeneity of Fe probably affected the outlier values of CR from the field next to the Mining Hill and legacy mines, possibly in addition to the differences in soil grain-size distribution. Across locations, the CR values of REE all appeared to have a positive bias from associated soil mass (as discussed above). Moreover, across locations, the difference between CRs for cereal species was also notable and could be assessed in future studies. However, since there have been few CR values of REE published, the ones from the present study make an important contribution to the underpinning datasets required for risk assessment. It is also worth noting that the present study provides background levels for NOR and REE in this area, given that planning and implementation of new mining activities are ongoing for this aera (Fen complex), as a baseline will be required to be able to consider any future contamination from such mining on soil or crops.

## Conclusions

Holocene deposits cover much of the high background bedrock of Fen complex and most agricultural soils and cereals are not particularly affected by underlying bedrock. However, especially one agricultural field has enhanced values of NOR and REE, and is probably impacted by HBRA soil and/or debris from legacy mining. A slight enhancement of REE in most of the Fen agricultural soil samples may be explained by input by weathering of local bedrock over geological time. The cereal from the outlier field impacted by the Mining Hill is the only one with significant NOR and REE levels. In this agricultural field, levels of NOR and REE in soil are very heterogenous, as are also cereal uptake. Notably, the CR values of REE in the present study are important contributions, as few such data have been previously published.

Like other authors we did not find any significant effect of washing the cereal, but we identified soil mass in cereal samples by using an acid solution that was optimized for dissolving both cereal and soil. If not using such an optimal acid combination, any bias from soil particles may be less conspicuous or not discovered, due to not dissolving the soil grains but only the cereal. However, such soil particles will contribute to dose if not removed before ingestion. We also estimated this soil mass and its bias using Ti and Sc as tracers but attained overestimates, particularly for Sc, probably due to a higher than anticipated root uptake.

## Data Availability

The data will be made available on acceptance of the publication in Mendely data repository . See text now for temporary doi.
